# Electrocautery, Harmonic, and Thunderbeat Instruments in Parotid Surgery: A Retrospective Comparative Study

**DOI:** 10.3390/jcm11247414

**Published:** 2022-12-14

**Authors:** Luigi Angelo Vaira, Davide Rizzo, Claudia Murrocu, Caterina Francesca Zullo, Margherita Dessy, Luca Mureddu, Enrica Ligas, Giovanni Salzano, Andrea Biglio, Miguel Mayo-Yáñez, Jerome R. Lechien, Pasquale Piombino, Francesco Bussu, Giacomo De Riu

**Affiliations:** 1 Maxillofacial Surgery Operative Unit, Department of Medicine, Surgery and Pharmacy, University of Sassari, 07100 Sassari, Italy; 2 Biomedical Science Department, PhD School of Biomedical Science, University of Sassari, 07100 Sassari, Italy; 3 Otorhinolaryngology Operative Unit, Department of Medicine, Surgery and Pharmacy, University of Sassari, 07100 Sassari, Italy; 4 Maxillofacial Surgery Operative Unit, University Hospital of Naples “Federico II”, 80131 Naples, Italy; 5 Otorhinolaryngology, Head and Neck Surgery Department, University Hospital Complex of A Coruña (CHUAC), 15006 A Coruña, Spain; 6 Department of Human and Experimental Oncology, Faculty of Medicine, UMONS Research Institute for Health Sciences and Technology, University of Mons (UMons), 7000 Mons, Belgium; 7 Department of Otolaryngology-Head Neck Surgery, Elsan Polyclinic of Poitiers, 86000 Poitiers, France

**Keywords:** parotidectomy, parotid, Harmonic, Thunderbeat, electrocautery, ultrasonic instruments, advanced bipolar coagulation, combined energy instruments, maxillo-facial surgery, otorhinolaryngology

## Abstract

The aim of this retrospective study has been to compare the surgical outcomes of patients undergoing superficial parotidectomy with three different instruments: bipolar electrocautery, ultrasound, and mixed energy instruments. The clinical records of 102 patients who had undergone superficial parotidectomy for benign tumors between January 2016 and April 2022 were considered. Based on the tool used during the surgery, the patients were divided into three study groups: classic electrocautery hemostasis group (CH group), ultrasonic instrument group (HA group), and combined energy instrument group (TB group). The duration of surgery, the total post-operative drainage volume, and the intra-operative blood loss were significantly higher in the CH group compared to the HA and the TB group, while the differences were not significant between the latter two groups. Facial nerve weakness was detected in 45.9% of the CH group, 12.5% of the HA group, and 21.2% of the TB group. The rate of facial nerve dysfunction in the CH group was significantly higher than in the HA group (0.011). In the patients who experienced post-operative facial nerve dysfunction, the recovery time was significantly shorter in the HA group compared to the CH and the TB group. The HA and TB groups have demonstrated comparable and significantly better surgical outcomes than bipolar electrocautery. Ultrasound instruments have been shown to cause, in comparison with the other techniques, a lower rate of temporary facial nerve dysfunction and, if this is present, lead to a faster spontaneous recovery time.

## 1. Introduction

Parotidectomy is one of the most frequently performed procedures in head and neck surgery departments [1]. Although routinely carried out, this procedure requires great attention during the dissection of the gland from the facial nerve in order to avoid temporary or permanent nerve deficits, which represent one of the most frequent complications of this type of surgery [2,3]. For this reason, over time, new tools have been proposed to achieve hemostasis during the dissection of the gland in an attempt to limit the traumatic damage to the surrounding tissues [4]. The classic electrocautery instruments develop high temperatures (i.e., 150–400 °C) in order to induce obliterative coagulation. All this heat can be transmitted to the surrounding glandular tissue, damaging the nerve [5,6].

Since the 1990s, ultrasonic instruments have been introduced. These tools are capable of cutting and coagulating tissues by converting electrical energy into ultrasonic vibrations, developing little heat (60–80 °C), which is not dispersed over more than 100 microns from the point of application [7]. For this reason, the use of ultrasonic instruments has been proposed for various surgical procedures in the head and neck district [8,9,10,11]. Some authors have investigated the efficacy and safety of ultrasonic instruments in parotid surgery [12,13,14,15,16,17,18,19]. The recent meta-analysis by Li et al. [20] concluded that ultrasound instruments, compared to classic electrocautery, guarantee better results in terms of operating time, intraoperative hemorrhage, hospitalization, salivary fistulas, and transient paralysis of the facial nerve.

More recently, new integrated energy instruments capable of combining ultrasonic energy with advanced bipolar coagulation have been proposed [21]. The combination of the two systems allows a limitation in the amount of energy by reducing the heat produced and its lateral diffusion, which is comparable to that of ultrasonic instruments [22]. The first reports in thyroid surgery have found a reduction in surgical times with integrated energy instruments compared to ultrasonic tools [23,24,25]. Furthermore, on animal models, combined energy instruments have shown the best efficacy and rapidity in sealing vessels [26].

However, to the best of our knowledge there are no reports on the results of using these tools in parotid surgery nor studies comparing the effectiveness of these three hemostasis techniques. Therefore, the aim of this retrospective study was to compare the surgical outcomes of patients undergoing superficial parotidectomy with three different instruments: bipolar electrocautery, ultrasound, and mixed energy instruments.

## 2. Materials and Methods

This retrospective study was conducted at the Head and Neck Surgery Department of the University Hospital of Sassari. Due to its retrospective nature, the study did not require any approval from the ethics committee and was conducted in accordance with the Helsinki declaration.

For the purposes of the study, the clinical records of patients who had undergone superficial parotidectomy for benign tumors between January 2016 and April 2022 were considered. All the operations had been performed by the same surgical team (i.e., first and second operator) with a long experience in parotid surgery.

Patients were excluded from the study if they had one of the following exclusion criteria: age < 18 years; previous parotid surgery; a history of acute or chronic sialadenitis, previous facial nerve weakness, neurological or psychiatric comorbidities, coagulation disorders, anticoagulant or antiplatelet therapy; ASA > 2; or incomplete data.

Some general data were retrieved and collected for all the patients: gender, age, size (i.e., maximum diameter detected on histological examination), histological diagnosis of the tumor, and type of surgical instrument used during surgery. The surgical outcomes considered were duration of the surgery, intra-operative complications, intra-operative blood loss, total fluid post-operative drainage, duration of the drains being kept in place, duration of hospitalization, presence and duration of facial nerve weakness, and post-operative complications (hemorrhage, hematoma, seroma, salivary fistulas, or Frey syndrome). Any intra-operative bleeding was estimated by means of a comparison between pre- and post-operative blood hemoglobin. Facial nerve function was assessed by means of the House-Brackmann scale [27], which classifies the degree of nerve function into 6 grades ranging from normal function (grade 1) to total paralysis (grade 6). This scale is routinely used in our center both during hospitalization and in post-operative follow-up.

**Figure 1:**
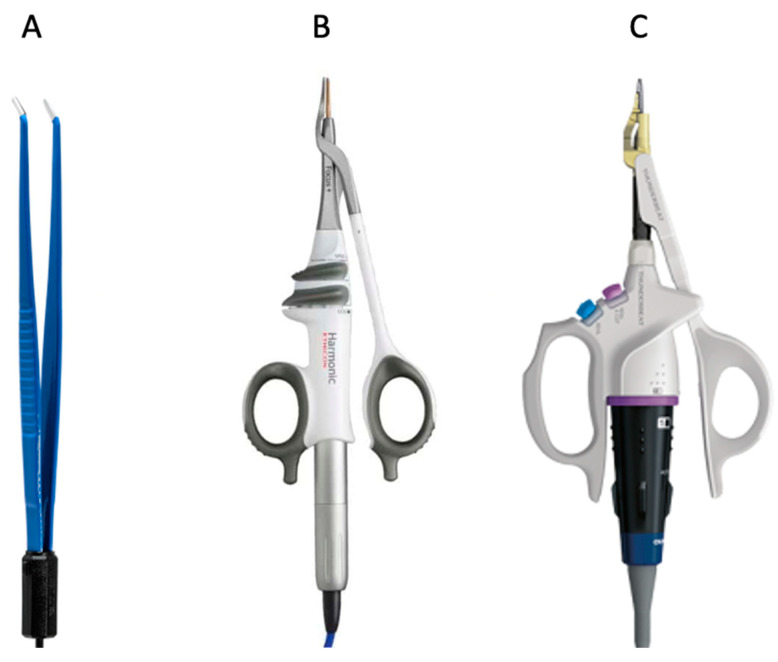
(**A**) Classic bipolar coagulator forceps used in the CH group. (**B**) Harmonic Focus Shears^®^ used in the HA group. (**C**) Thunderbeat open fine jaw^®^ used in the TB group.

Based on the tool used during the surgery (Figure 1), the patients were divided into three study groups:

1. Classic electrocautery hemostasis group: patients operated on with bipolar forceps (Aesculap Inc., Center Valley, PA, USA) between January 2016 and October 2018 (the CH group).
2. Ultrasonic instrument group: patients operated on with Harmonic Focus Shears^®^ (Ethicon Inc., Raritam, NJ, USA) between November 2018 and April 2020 (the HA group).
3. Combined energy instrument group: patients operated on with Thunderbeat open fine jaw^®^ (Olympus Medical Systems Corp., Tokyo, Japan) between May 2020 and April 2022 (the TB group).

### 2.1. Surgical Procedure

In accordance with the diagnostic protocol of the center, all the patients had been subjected to a pre-operative evaluation with CT or MRI with contrast medium and fine needle aspiration of the tumor. Once the suspicion of a benign superficial parotid tumor had been established, the patients underwent surgery under general anesthesia. A modified Blair skin incision was performed in all cases. During the elevation of the skin flap, hemostasis was obtained using the same instrument then used for the parotid dissection. After identifying the main trunk of the facial nerve, the superficial parotid was detached and excised, including the tumor. The dissection of the nerve branches, starting from the main trunk, was carried out using a Mixter clamp. Once separated from the nerve, the gland was divided and sealed with bipolar forceps and scissors, Harmonic scalpel, or Thunderbird, as appropriate. Finally, a suction drain was placed and the skin was closed in layers.

### 2.2. Statistical Analysis

Statistical analyses were performed using Jamovi version 2.3.18.0, a freeware and open statistical software available online at www.jamovi.org (accessed on 2 November 2022) [28]. Categorical variables are reported in numerals and percentages of the total. Descriptive statistics for quantitative variables are given as the median [interquartile range (IQR)] or mean ± standard deviation (SD). The Chi2-square test and one-way ANOVA were performed to evaluate the differences between the three groups in terms of categorical and continuous variables, respectively. In any case of significance of the one-way ANOVA the post-hoc analysis was carried out with the Games-Howell test in any case of different variance between the groups, or with the Tukey test, in any case where the variances were the same. The level of statistical significance was set at *p* < 0.05 with a 95% confidence interval.

## 3. Results

Between January 2016 and April 2022, 177 patients underwent a superficial parotidectomy for benign tumor resection at the University Hospital of Sassari. Of these, 75 patients were excluded from this analysis for the following reasons: age < 18 years (1 patient), a history of acute or chronic sialadenitis (2 patients), previous facial nerve weakness (1 patients), neurological or psychiatric comorbidities (8 patients), coagulation disorders or anticoagulant or antiplatelet therapy (18 patients), ASA > 2 (23 patients), and incomplete data (24 patients).

102 patients who met the inclusion criteria were then included in the study and divided into the three study groups: the CH group with 37 patients, the HA group with 32 patients and the TB group with 33 patients. The three groups were homogeneous for gender, age, size, and histology of the tumor [Table 1].

**Figure 2:**
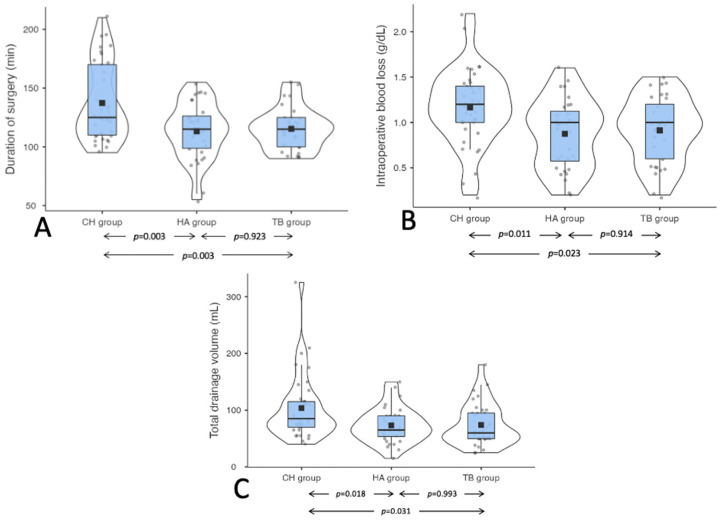
(**A**) Duration of surgery differences between the groups. (**B**) Intraoperative differences between the group. (**C**) Total drainage volume differences between the groups. Legend: CH Classic bipolar hemostasis group; HA: Harmonic group; TB: Thunderbeat group.

The duration of surgery was significantly higher in the CH group compared to the HA group (125 min [IQR 60] versus 115 min [IQR 27.5]; *p* = 0.003) and the TB group (125 min [IQR 60] versus 115 min [IQR 25]; *p* = 0.003). No significant differences were found between the latter two groups (*p* = 0.923) (Figure 2A). Intra-operative blood loss, estimated in terms of the difference between pre- and post-operative hemoglobin, was significantly greater in the CH group than in the HA group (−1.2 g/dL [IQR 0.4] versus −1 g/dL [IQR 0.55]; *p* = 0.011) and the TB group (−1.2 g/dL [IQR 0.4] versus −1 g/dL [IQR 0.6]; *p* = 0.023), while the differences were not significant between the latter two groups (*p* = 0.914) (Figure 2B). The total post-operative drainage volume was also significantly higher in the CH group than in the HA group (85 mL [IQR 45] versus 65 mL [IQR 36.3]; *p* = 0.018) and the TB group (85 mL [IQR 45] versus 60 mL [IQR 45]; *p* = 0.031), with no significant differences between the latter two groups (*p* = 0.993) (Figure 2C).

**Figure 3:**
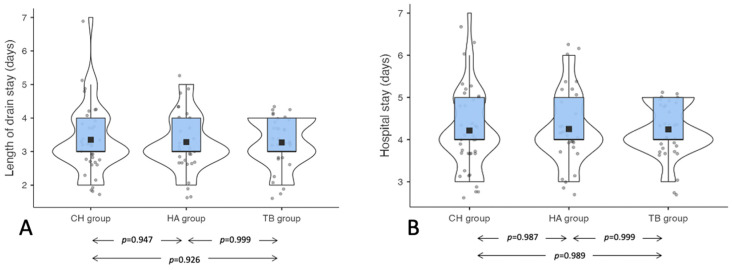
(**A**) Length of drain stay differences between the groups. (**B**) Length of hospital stay between the group. Legend: CH: Classic bipolar hemostasis group; HA: Harmonic group; TB: Thunderbeat group.

No significant differences were found between the groups in terms of the time required for the drainage removal and the duration of hospitalization (Figure 3A,B).

The three study groups did not present any significant differences in terms of post-operative complications such as hematomas, hemorrhages, seromas, salivary fistulas, and Frey syndrome [Table 2].

**Figure 4:**
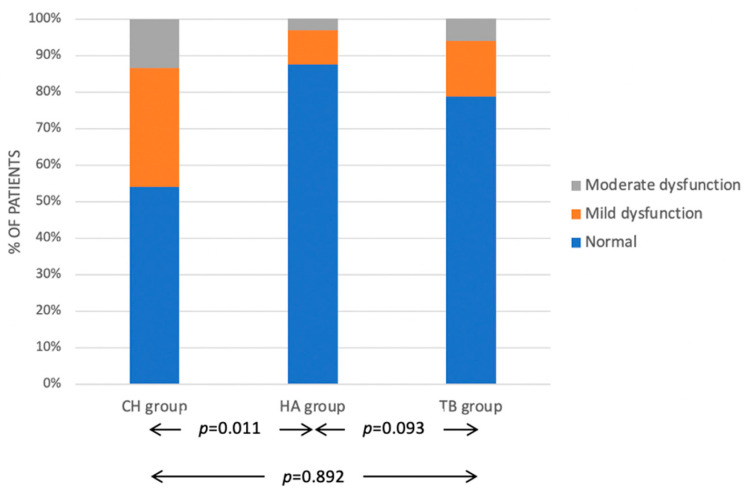
Facial nerve function in the three study groups according to of the House-Brackmann scale [27]. Legend: CH Classic bipolar hemostasis group; HA: Harmonic group; TB: Thunderbeat group.

An intraoperative section of the facial nerve or one of its branches has not been reported in any case. Facial nerve weakness was detected in 45.9% of the CH group (including mild dysfunction in 12 cases and moderate dysfunction in 5 cases), 12.5% of the HA group (including mild dysfunction in 3 cases and moderate dysfunction in 1 case) and 21.2% of the TB group (including mild dysfunction in 5 cases and moderate dysfunction in 2 cases). The rate of facial nerve dysfunction in the CH group was significantly higher than in the HA group (0.011). The differences were instead non-significant between the CH and the TB group (*p* = 0.093) and between the HA and the TB group (*p* = 0.892) (Figure 4).

**Figure 5:**
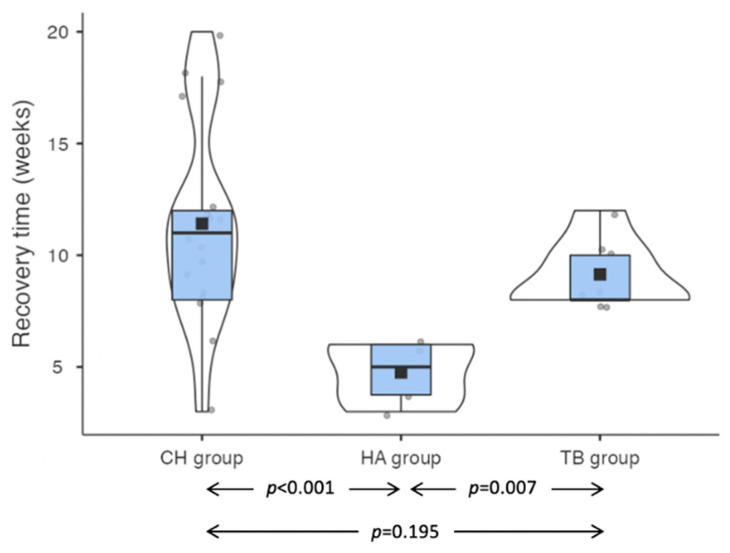
Facial nerve recovery times in the three study groups. Legend: CH: Classic bipolar hemostasis group; HA: Harmonic group; TB: Thunderbeat group.

In the patients who experienced post-operative facial nerve dysfunction, the recovery time was significantly shorter in the HA group compared to the CH group (5 weeks [IQR 2.25] versus 11 weeks [IQR 4]; *p* < 0.001) and the TB group (5 weeks [IQR 2.25] versus 8 weeks [IQR 2]; *p* = 0.007). No significant differences were found between the CH group and the TB group (*p* = 0.195) (Figure 5). In all cases, nerve recovery was complete.

## 4. Discussion

The parotid gland is densely vascularized and the control of hemostasis is crucial during parotidectomy in order to prevent hemorrhage and hematoma. The vascular network is closely related to the facial nerve and hemostasis procedures are essential to maintain a clear view of the surgical field, but these can cause iatrogenic nerve dysfunction [29]. Moreover, reducing intra- and post-operative blood loss results in a shorter recovery time and, therefore, lower healthcare costs [30]. Finally, during partial parotidectomies it is essential to obtain an adequate sealing of the residual glandular lobules in order to avoid salivary fistulas [31].

Bipolar electrocauteries represent the workhorse in the control of hemostasis in parotid surgery and allow an effective sealing of vessels up to 7 mm in diameter [32]. However, the high heat developed during their use can damage the surrounding tissues and this can lead the surgeon to be less attentive to hemostasis in order to avoid damage to the facial nerve [4]. The state-of-the-art form of these instruments is the advanced bipolar coagulation, which ensures the same performance in sealing the vessels while maintaining temperatures lower than 100 °C even if used for a prolonged time [33,34].

Ultrasonic instruments have been proposed to overcome some of the limitations of electrocauteries as they develop lower temperatures and, unlike the latter, allow vessels and tissues to be sealed and cut at the same time [9]. However, if used at a high power and for a prolonged time, they can still reach temperatures above 100 °C with the risk of damage to the surrounding structures [35,36]. In recent years, mixed energy instruments have been recommended to try to exploit the advantages of both instruments (i.e., the ability of ultrasonic instruments to simultaneously seal and cut tissues and the low temperatures associated with advanced bipolar coagulation even for long applications) [21,37].

This study has the advantage of comparing the efficacy and safety of these three types of instruments in parotid surgery. Ultrasonic and combined energy tools have demonstrated significantly better results than bipolar tools in terms of duration of surgery, and intra- and post-operative blood loss. This is related to a greater safety of these tools in sealing the vessels without stimulating the nerve, even at a very close proximity. These findings are in line with those previously observed for ultrasonic instruments in parotidectomies [12,13,14,15,16,17,18,19,20] and for combined energy instruments in thyroid surgery [24,25,30]. However, the main non-neurological post-operative complications did not reveal any significantly different prevalence between the study groups. This is in line with the results reported by other authors in comparative studies between electrocautery and ultrasonic instruments [13,38]. Only Deganello et al. [18] found a higher rate of Frey syndrome in patients operated on with bipolar instruments, but this finding was not corroborated by the meta-analysis by Li et al. [20]. It is probable that the onset of Frey syndrome is mainly linked to other factors such as the use of an interposition barrier [39]. Compared to bipolar electrocautery, ultrasonic instruments have demonstrated a significantly lower rate of transient facial nerve dysfunction. Furthermore, the speed of nerve dysfunction recovery was significantly faster than with both bipolar and combined energy instruments. This finding is in line with that reported by other authors [12,13,14,15,16,17,18,19] and is correlated to the lesser damage of surrounding tissues [20] caused by the ultrasonic instruments and the shorter duration of the surgery [40]. The combined energy tools showed no significant differences in terms of facial nerve deficits compared to both other groups. The recovery time was significantly longer than with ultrasonic instruments and did not differ significantly from the CH group. A higher rate of transient recurrent laryngeal nerve dysfunction with combined energy tools than with ultrasonic tools has been previously reported [24,25]. Both instruments work at similar temperatures, do not differ in lateral heat diffusion, and can be used safely at least 2 mm from the nerve and for a maximum of 8 consecutive seconds [34,35]. It is possible that the difference found lies in the conformation of the instrument. The Thunderbeat, which has been proposed for general and endoscopic surgery, has a relatively large and blunt cutting blade compared with the Harmonic focus, and can therefore be unwieldy in delicate operations such as parotid surgery [41]. It would be important in the future to have combined energy instruments specifically designed for head and neck surgery.

This study has some limitations which need to be acknowledged. First, it is not randomized and has included patients who have been operated on with different instruments over time, chosen on the basis of the availability offered by the hospital. However, the study groups did not present any significant differences in epidemiological and clinical characteristics. Secondly, the evaluation of the facial nerve deficit has been performed by different surgeons and, although the classification of House-Brackmann is standardized and validated, it is based on a subjective evaluation. In the future, it will be important to investigate this aspect through nerve monitoring. Thirdly, the sample size was sufficient to detect some strong and statistically significant correlations between the type of instrument used and the surgical outcomes. Nevertheless, it is possible that we might have rejected some associations due to a type 2 statistical error.

## 5. Conclusions

The ultrasonic and the combined energy instruments have demonstrated comparable and significantly better surgical outcomes than bipolar electrocautery. Ultrasound instruments have been shown to cause, in comparison with the other techniques, a lower rate of temporary facial nerve dysfunction and, if this is present, lead to a faster spontaneous recovery time.

## Figures and Tables

**Table 1:** The patients’ general characteristics.

	CH Group (*n* = 37)	HA Group (*n* = 32)	TB Group (*n* = 33)	*p*-Value
**Gender** *n* (%)				
Male	16 (43.2%)	16 (50%)	18 (54.5%)	0.635 *
Female	21 (56.8%)	16 (50%)	15 (45.5%)	
**Age (years)** Mean ± SD	54.8 ± 15.6	57.1 ± 14.9	55.9 ± 12.5	0.815 **
**Tumor size (cm)** Mean ± SD	2.27 ± 1.14	2.31 ± 1.13	2.44 ± 0.95	0.769 **
**Tumor type** *n* (%)				
Pleomorphic adenoma	28 (75.7%)	21 (65.6%)	23 (69.7%)	0.792 *
Warthin tumor	9 (24.3%)	10 (31.3%)	9 (27.3%)	
Oncocytoma	0 (0%)	1 (3.1%)	1 (3%)	

Legend: * Chi2 test; ** One-way ANOVA.

**Table 2:** Complications rate analysis.

Complication *n* (%)	CH Group (*n* = 37)	HA Group (*n* = 32)	TB Group (*n* = 33)	*p*-Value
Salivary fistula	3 (8.1%)	1 (3.1%)	3 (9.1%)	0.593 *
Hemorrhage	0 (0%)	0 (0%)	0 (0%)	1 *
Hematoma	1 (2.7%)	0 (0%)	1 (3%)	0.624 *
Seroma	0 (0%)	0 (0%)	0 (0%)	1 *
Frey syndrome	2 (5.4%)	1 (3.1%)	0 (0%)	0.408 *
Facial nerve weakness	17 (45.9%)	4 (12.5%)	7 (21.2%)	0.005 *

Legend: * Chi2 test.

## Data Availability

L.A.V. and G.D.R. have full access to data reported in this study.
